# Bactericidal, Virucidal, and Biocompatible Properties of 3D Printed Materials Enhanced with Copper and Zinc Nanoparticles

**DOI:** 10.1002/gch2.202500106

**Published:** 2025-06-16

**Authors:** Andrei‐Florin Sandu, Lauren Acton, Phillip Gould

**Affiliations:** ^1^ Coventry University Group: Coventry University Warwickshire 2706 UK

**Keywords:** 3D printing, bactericidal, biocompatible, nanoparticles, virucidal

## Abstract

The heightened spread of pathogens due to population growth, urbanization, and climate change presents significant health challenges, exacerbated by high transmission, virulence, antimicrobial resistance (AMR), and novel variants. Hospital‐acquired infections (HAI) affect 1 in 31 hospitalized patients, costing $28.4 billion annually. This study introduces a novel approach to pathogen control by integrating copper and zinc oxide nanoparticles into 3D printed Stereolithography (SLA) materials. The 3D impregnated material demonstrates reproducibility and efficacy across different 3D platforms, showcasing complete bactericidal/fungicidal effects against twelve diverse species and a 4 log virucidal activity on eight clinically relevant viral species within 2 h. No significant cytotoxicity is observed in primary human keratinocytes after 2 h of contact. The material maintains its antipathogenic activity after a year of accelerated ageing, suggesting enhances stability and performance over time. This method addresses the limitations of conventional cleaning and surface spraying, which often fall short in efficacy and longevity; for the first time, the incorporation of commercially available nanoparticles into 3D printable materials offers a versatile long‐lasting antipathogenic and biocompatible solution for high‐contact surfaces in public and clinical settings, reducing the need for cleaning surfaces while limiting infection rates, the threat of AMR, and other future infectious outbreaks.

## Graphical abstract

As population growth, urbanization, and climate change increase the spread of pathogens, hospital infections now affect 1 in 31 patients, costing billions annually. This study presents a new approach by integrating copper and zinc oxide nanoparticles into 3D‐printed materials. This material effectively neutralizes pathogens while remaining biocompatible and stable for over a year, offering a long‐term solution for infection control.

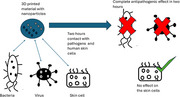

## 1 Introduction

Population growth, urbanization, climate change, and transport development have accelerated pathogen spread and global transmission, creating infectious hotspots in urban areas.^[^ ^1^, ^2^ ^]^ This was exemplified by SARS‐CoV‐2, which spread to multiple countries within two months of its emergence.^[^ ^3^ ^]^ Shifting population growth and dynamics in urban areas, combined with factors like poor water storage and overcrowding, facilitate pathogen transmission, leading to outbreaks such as cholera;^[^ ^4^ ^]^ additionally, the possible adaptation of disease vectors, such as the malaria‐carrying *Anopheles* mosquito, further exacerbates the risk of disease spread.^[^ ^5^ ^]^ Climate change can drive pathogen mutation, leading to the emergence of novel variants, enhanced virulence, and outbreaks;^[^ ^6^, ^7^, ^8^ ^]^ Additionally, the expansion of global trade and transportation has accelerated the spread of pathogens across ecological and geographical boundaries, promoting adaptation in new environments as observed for Zika and dengue virus.^[^ ^9^, ^10^ ^]^ These factors raise health concerns related to antimicrobial resistance (AMR), enhanced transmission, and high virulence.

Rapid transmission of pathogens driven by urbanization and increased global mobility is evident in recent outbreaks.^[^ ^11^ ^]^ For instance, SARS‐CoV‐2 infected ≈775 million people globally and caused over 7 million deaths from December 2019 to August 2024.^[^ ^12^ ^]^ Similarly, the 2022/2023 flu season resulted in ≈97 000 deaths,^[^ ^13^ ^]^ with annual influenza cases ranging from 9 to 45 million and fatalities from 12 000 to 61 000 since 2010,^[^ ^13^ ^]^ specifically, the 2009 H1N1 pandemic caused an estimated 151 700 to 575 400 deaths in its first year.^[^ ^14^ ^]^ A similar pattern can be identified in hospitals, where pathogen transmission is heightened by patient movement, as observed for multidrug‐resistant *Enterobacteriaceae* and viruses like human parainfluenza, and in neonatal units by rhinovirus and RSV.^[^ ^15^, ^16^, ^17^, ^18^ ^]^ Nosocomial infections and AMR affect 5–10% of patients and healthcare workers annually, costing over $28.4 billion.^[^ ^19^ ^]^

Despite strategies like water treatment, immunization, and hygiene, outbreaks of bacterial, fungal, and viral infections persist.^[^ ^20^ ^]^ Pathogen prevention relies on cleaning with detergents, disinfectants, UV sterilization, and automated devices like hydrogen peroxide aerosolization, but often remains inadequate.^[^ ^21^, ^22^, ^23^ ^]^ For example, bacterial infections, including Meticillin‐resistant *Staphylococcus aureus* (MRSA), remain a significant health threat, with MRSA causing 31.8 cases per 1 00 000 people and 6.2 hospital‐acquired infections (HAIs) per 1000 admissions.^[^ ^24^, ^25^ ^]^ In 2018, 1.7 million HAIs in the US led to over 98 000 deaths.^[^ ^26^ ^]^ Daily hospital cleaning often falls short; 50% of environments remain uncleaned after routine housekeeping.^[^ ^27^ ^]^ Bacterial isolates from common hospital items show that 46% are pathogenic,^[^ ^28^ ^]^ underscoring the need for better infection control strategies, as U.S. facilities spend over $2.6 million annually on general housekeeping.^[^ ^29^ ^]^

It is predicted that by 2050, antibiotic resistance could result in 10 million deaths and a $100 trillion global economic burden.^[^ ^30^ ^]^ Given the severe threat of AMR and the unsatisfactory sanitation efficiency,^[^ ^27^, ^30^ ^]^ effective measures such as using materials that reduce infection transmission without antibiotic usage are crucial.^[^ ^31^ ^]^ Antimicrobial coatings can effectively reduce pathogen contamination and lower infection rates, directly reducing AMR, housekeeping time, and financial strain.^[^ ^32^, ^33^, ^34^ ^]^ Studies show that material coatings can reduce bacterial contamination by up to 98% for *S. epidermidis*,^[^ ^35^ ^]^ and achieve over 60% pathogen reduction in hospital settings,^[^ ^32^, ^36^, ^37^ ^]^ with more than 40% reduction in nosocomial infections even against MRSA.^[^ ^34^, ^38^, ^39^ ^]^ The effectiveness of antimicrobial coatings is dependent on the materials and agents used.^[^ ^40^ ^]^ For example, passive coatings prevent bacterial attachment or kill bacteria on contact, with surface characteristics like roughness, wettability, and conductivity influencing bacterial behavior.^[^ ^41^ ^]^ Active coatings, on the other hand, release antibacterial agents such as antibiotics and bioactive molecules.^[^ ^41^ ^]^ Both antipathogenic coating strategies overall offer a more durable protection than traditional sterilization methods due to the long‐lasting effects and minimal maintenance.^[^ ^40^, ^42^ ^]^

The antimicrobial properties of nanoparticles against both gram‐positive and gram‐negative bacteria present a promising avenue in combating pathogenic spread and AMR.^[^ ^43^ ^]^ Copper nanoparticles present enhanced characteristics such as high surface area and catalytic activity; they are used in electronics, catalysts, biomedical applications, and as antimicrobial agents.^[^ ^44^, ^45^ ^]^ They effectively reduce pathogens by damaging cell membranes, generating Reactive Oxygen Species (ROS), and interfering with DNA replication.^[^ ^46^ ^]^ Similarly, zinc oxide nanoparticles are used in water treatment, sunscreens, electronics, and cosmetics, offering diverse applications due to their photocatalytic and UV absorption properties.^[^ ^47^, ^48^, ^49^ ^]^ They exhibit neutralization strategies through ROS generation, oxidative stress, membrane and DNA damage, Zn^2+^ ion release, and enzyme inhibition.^[^ ^50^ ^]^ While metal nanoparticles are effective against bacteria and viruses,^[^ ^51^, ^52^ ^]^ they can also be toxic to mammalian cells.^[^ ^53^ ^]^ Therefore, developing versatile antimicrobial materials by use of nanoparticles is crucial to address both current and future pathogen threats amid increasing urbanization and environmental changes. However, rigorous testing is essential to prevent the release of cytotoxic or nano‐toxic effects.^[^ ^34^ ^]^

While it is hypothesized that pathogens might evolve resistance mechanisms to surface coatings, there is very limited evidence that has been observed.^[^ ^54^ ^]^ Prolonged exposure to toxic elements can lead to mutations and resistance, as seen with *Staphylococcus aureus* developing silver resistance.^[^ ^55^, ^56^ ^]^ Bacteria and fungi, like *Candida albicans*, use survival strategies such as biofilm formation to protect against toxic environmental elements.^[^ ^57^ ^]^ Bacteria employ various tactics, including ion charge changes and bioleaching, to survive.^[^ ^58^, ^59^ ^]^

During the COVID‐19 pandemic, 3D printing was used to develop protective visors and N‐95 masks, addressing critical shortages and demonstrating its adaptability and impact.^[^ ^60^, ^61^, ^62^ ^]^ Additive manufacturing technology has revolutionized industries with its low cost and rapid production capabilities, offering extensive customization. The variety of materials available allows for functional and cost‐effective generation of prototypes, spare parts, and engineered items. Integrating 3D‐printed antipathogenic materials in hospitals and crowded places could offer better protection against airborne and fomite vectors by neutralizing pathogens.^[^ ^58^ ^]^ The potential of 3D‐printed virucidal materials is promising, providing rapid production without additional economic burden.^[^ ^63^, ^64^ ^]^

### 1.1 Aims

Nanoparticles offer vast applications in biology and could revolutionize 3D printing with their versatile properties. This research aims to evaluate and assess multiple nanoparticles and mixtures of nanoparticles, such as zinc, copper, silver, titanium, magnesium, bismuth, platinum, and optimize the integration of the best candidate mixture of nanoparticles into 3D printable Stereolithography (SLA) materials. The focus will be on overcoming challenges related to material incorporation by developing an easy, replicable, and straightforward method using commercially available materials, aiming to generate a biocompatible 3D‐impregnated material which will offer strong, versatile antipathogenic effects, according to ISO standards.

## 2 Results

### 2.1 Nanoparticles Selection

To determine the optimal active range of copper, zinc, and magnesium nanoparticles for achieving effective antimicrobial inhibition, each nanoparticle was first diluted in the appropriate solution and tested individually against each pathogen before progressing to the plastic impregnation stage (Figure S2, Supporting Information).

Further evaluation of nanoparticle combinations revealed enhanced activity in the copper‐zinc pairing (Figure S3, Supporting Information). Specifically, the copper‐zinc combination at an average of 1.12 mg mL^−1^ demonstrated the highest efficacy and was subsequently incorporated into a 3D material for further assessment of its antipathogenic properties. This modified material was designated as Material A.

### 2.2 Agar Diffusion Assay

**Figure 1:**
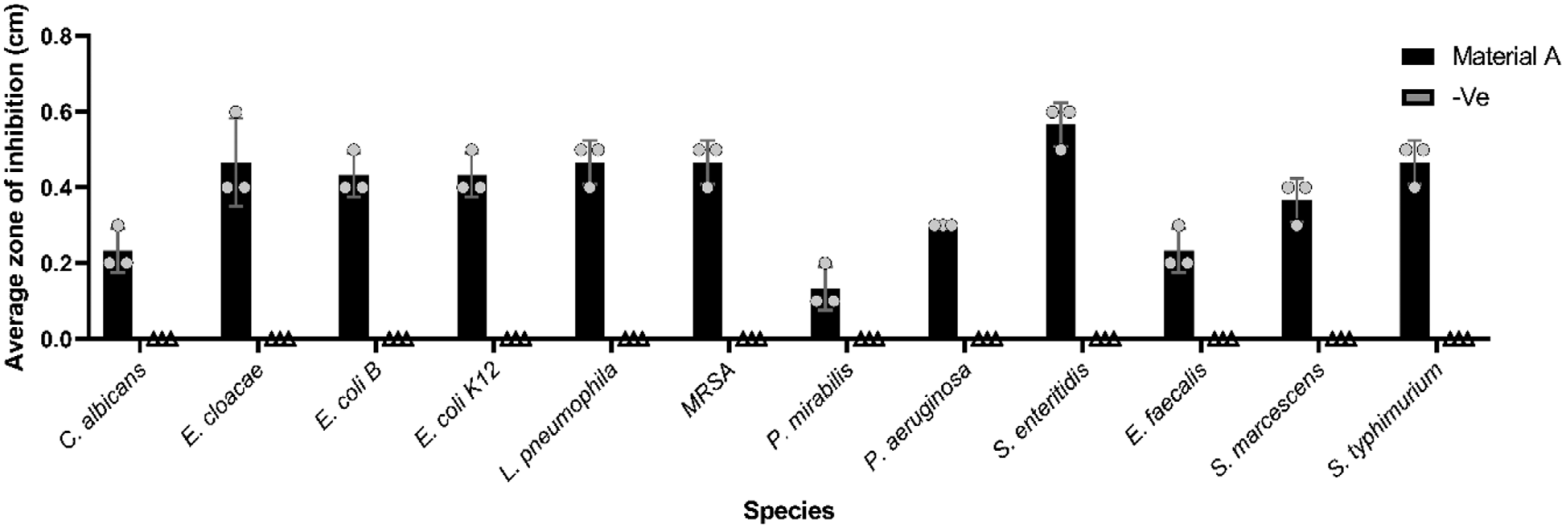
Agar diffusion results for Material A. The measurement of the inhibition zone created by the 3D impregnated material is represented in cm, with mean SD (n = 3).

All species tested (**Figure** **1**) showed direct inhibition by Material A (Figure S4, Supporting Information), the smallest inhibition zone recorded was for *P. Mirabilis* with an average of 0.13 cm (SD = 0.05) and the largest zone of inhibition was established by *S. enteritidis* with an average of 0.56 cm (SD = 0.05) (Figure 1). The control 3D material showed no zone of inhibition for any of the species tested (Figure S4, Supporting Information).

#### 2.2.1 Measurement of Antibacterial/Antifungal Activity on Plastics and Other Non‐Porous Surfaces ISO 22196:2011

Material A exhibits complete bactericidal and fungicidal effects against all tested species, with no viable growth observed, whereas the control material shows direct specimen growth. The bacterial/fungal stability test, performed according to ISO 22196:2011, revealed no values exceeding 0.2 after 18 h of incubation (Table S6, Supporting Information). This demonstrates that bacterial and fungal reduction is entirely reliant on the activity of the nanoparticles, with any decrease in microbial count attributed to the influence of Material A.

#### 2.2.2 Time Frame of Neutralization via Direct Contact for Bacteria and Fungi

**Figure 2:**
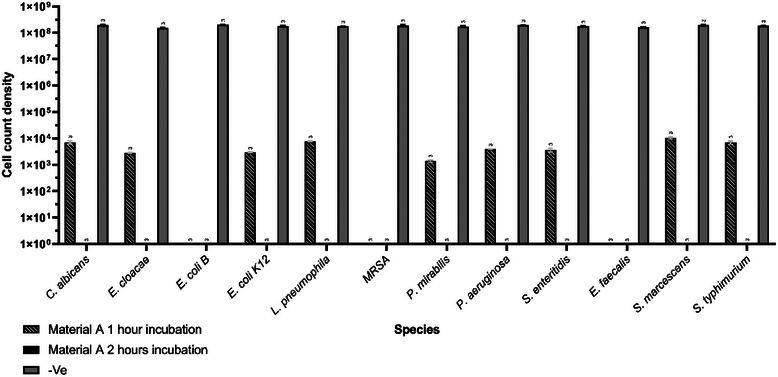
Time points optimization of direct contact testing. Direct contact material testing after 1 h and 2 h of incubation of 0.5 MFU bacterial count on material A and negative control (n = 3), mean with SD.

The 1 h time point of incubation of bacterial/fungal species with material A shows up to a 5‐log reduction with complete bactericidal effects for MRSA, *E. faecalis*, and *E. coli* B (**Figure** **2**).

Material A exhibited complete bactericidal and fungicidal effects against all tested species after 2 h of incubation (Figure 2), whereas the control material presented direct growth for all the species tested (Figure 2).

### 2.3 Antiviral Testing

Titers for all viral species were determined using the plaque assay protocol and optimized according to BS EN ISO 21702:2019.

**Figure 3:**
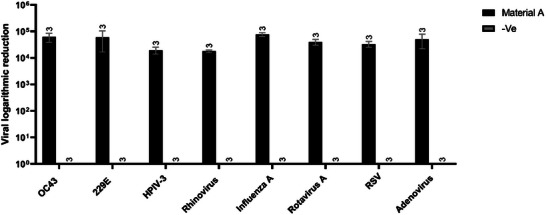
Plaque assay results (n = 3) after 2 h of contact of a viral specimen with material A and the negative control, represented as mean ± SD. All viruses present a titer reduction after 2 h of exposure to material A, and no viral reduction was observed for the negative control.

After 2 h of contact, Material A demonstrated more than four‐log viral reduction efficiency for all viruses tested, which indicates exceptional virucidal activity (BS EN ISO 21702:2019), whereas, in contrast, the 3D control material showed no viral reduction (**Figure** **3**).

### 2.4 Material Ageing

**Figure 4:**
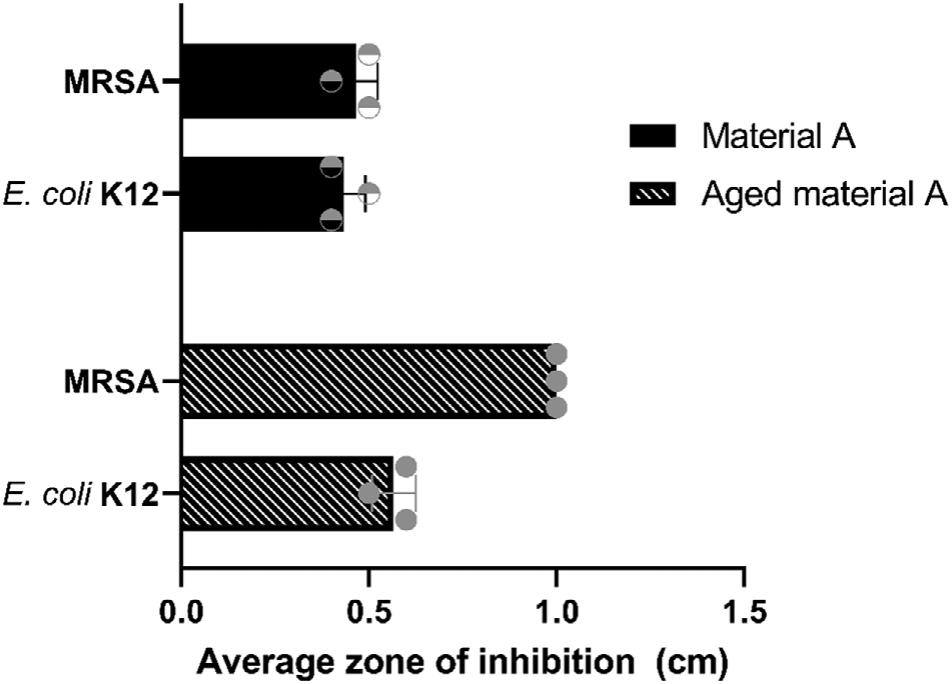
3D impregnated material ageing average zone of inhibition via agar diffusion with SD (n = 3). Results were represented in cm for *E. coli* K12 and MRSA against the one‐year‐old material. The error bars denote the standard deviation (SD = 0.06) difference between the material activities.

The materials did not present any physical or conformational changes via the accelerated ageing process (data not presented). The agar diffusion test (Figure S5, Supporting Information) shows that material A retains the antibacterial characteristics (**Figure** **4**), with enhanced inhibition against MRSA species (the new material has an area of 0.46 cm inhibition, whereas for aged material has 1 cm inhibition).

The virucidal testing against influenza A showed 4.6 logs of viral neutralization, indicating that material ageing did not affect the material virucidal capability, demonstrating a prolonged antipathogenic effect with long shelf‐life application.

### 2.5 Cytotoxicity

#### 2.5.1 Indirect Cytotoxicity

Cytotoxicity was tested using the human primary keratinocyte cell line (HEKa) following BS EN ISO 10993–5:2009. This in vitro test assesses cell monolayer reactivity, where grade 0 indicates no cytotoxicity and grade 4 indicates severe cytotoxicity. Distel, which is a commercial detergent, was used as a positive control. After 2 h of incubation, the positive control exhibited a severe toxic effect on the HEKa cell monolayer, with an average reactivity grade of 3.66 (near complete death).

**Figure 5:**
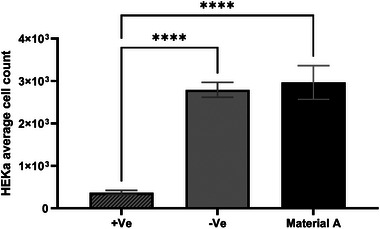
HEKa average cell count with SD for 2 h of contact via the indirect agar diffusion method (n = 3). The positive Distel control shows a significant difference (*p* < 0.0001) in cell count compared with all the 2 materials based on the one‐way ANOVA test, demonstrating that material A does not have significant cytotoxicity in 2 h of contact.

A cell count confirmed that the positive control significantly reduced total cell number (**Figure** **5**). Material A preserved cell confluence and monolayer integrity, with a reactivity grade of 1 (non‐toxic, bio‐compatible rating), showing no reduction in cell viability, and similar cell count to the inert negative control (3D printed disk without nanoparticles) (Figure 5). These findings suggest that a 2 h exposure to Material A does not induce toxic effects in human primary adult keratinocytes.

#### 2.5.2 Confocal Analysis for Cytotoxicity

Keratinocytes, the primary cell type in the epidermis, play a crucial role in forming the skin's barrier and defending against environmental stress. Confocal 2D and 3D analysis were conducted to assess whether Material A impacts the conformational structure and morphology of keratinocytes, as well as its potential effects on cell metabolism and the protective layers of these cells.

**Figure 6:**
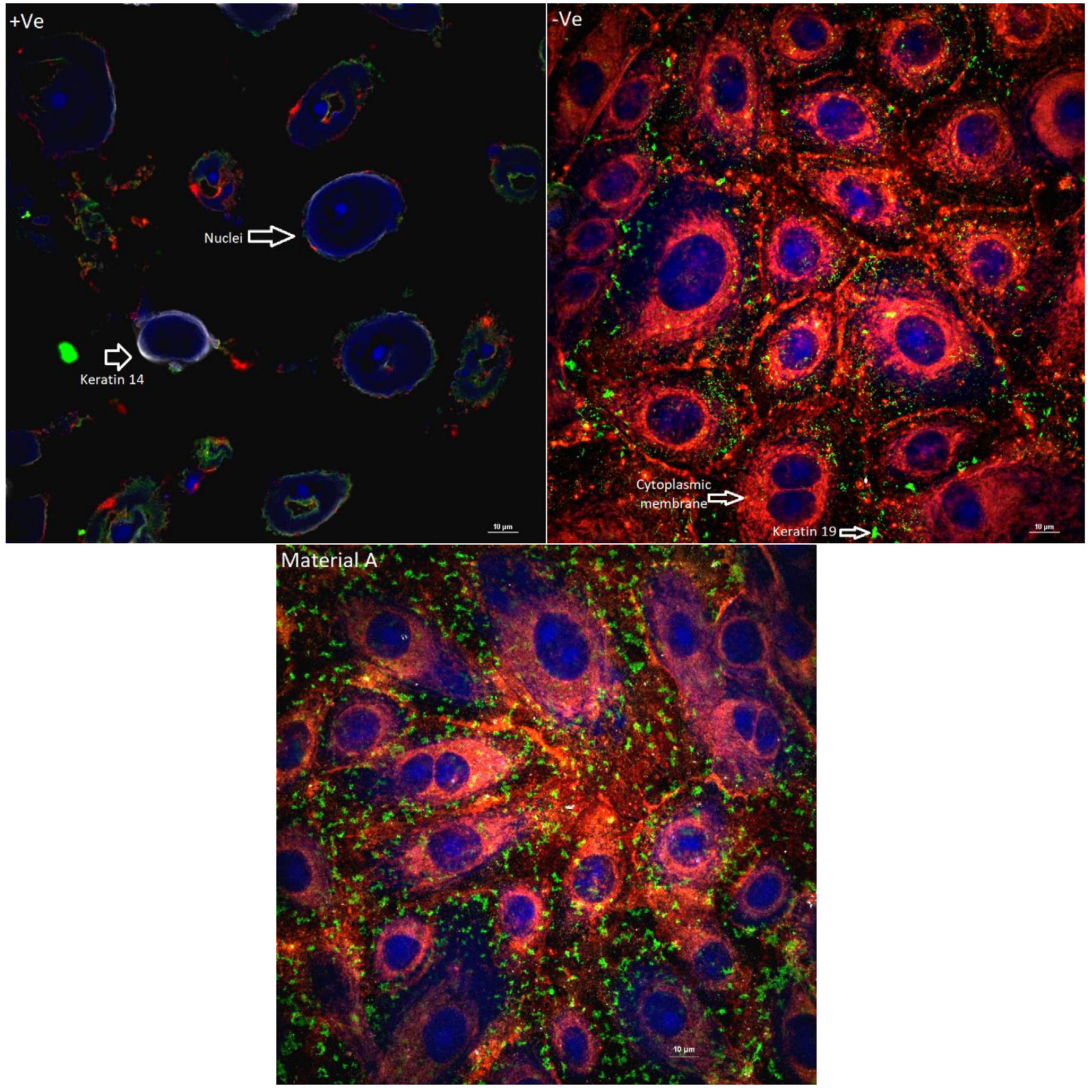
The cytotoxic effects were assessed using an indirect agar contact method (n = 3). The cytotoxic effects were visualized using confocal microscopy at 100x magnification. Human adult epidermal keratinocytes (HEKa) were stained: cytoplasmic membranes (red–orange), nuclei (blue), Cytokeratin 14 (white), and Cytokeratin 19 (green). Negative control showed normal morphology, while positive control showed abnormal cell structure. Material A displayed no cytotoxic effects, similar to the negative control.

The inert negative control (3D material without nanoparticles) exhibited no morphological alterations, maintaining a round shape with well‐defined borders (**Figure** **6**). Nuclei showed slight staining due to paraformaldehyde activation and cell membrane penetration, with a minimal distribution of keratin 14 and a uniform distribution of keratin 19 (Figure 6). In contrast, the positive control, after 2 h of incubation with Distel, displayed abnormal morphology, with hallows in the center of the cell, characterized by bright nuclei and a thin cellular membrane, accompanied by reduced keratin 19 and increased distribution of keratin 14 (Figure 6). Material A presents no cell damage, results and morphology similar to the negative control, with well‐defined cytoplasmic membrane borders, nuclei, and networks of keratin (Figure 6). These findings show that on a structural and morphological level, material A does not have a cytotoxic effect after 2 h of exposure, and the results from this method are identical with the indirect cytotoxic agar diffusion test.

**Figure 7:**
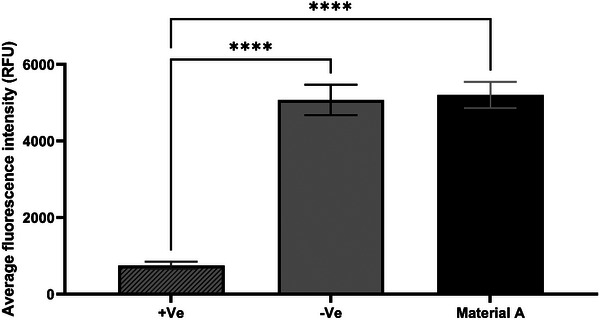
Average fluorescence intensity (RFU) for the 2 h of indirect contact cytotoxicity assay in primary adult human keratinocytes with SD (n = 3). Material A shows a similar RFU with the negative control and 3D control material, without any cytotoxic effect. The positive control is significantly different from material A according to the one‐way ANOVA test with a *p*‐value lower than 0.0001.

To ensure there is no discrepancy in the visual qualitative analysis, the average intensity of each image was established, which showed no significant difference between material A and the inert negative control (**Figure** **7**).

#### 2.5.3 3D Confocal

Keratins 14 and 19 are key proteins involved in maintaining keratinocyte integrity, stress resistance, and cell signaling. Keratin 14 supports cell shape, mechanical stress resistance, and apoptosis regulation, while Keratin 19 aids in adhesion, cytoskeleton organization, and stress protection. The presence or absence of these keratins in 3D confocal analysis helps evaluate the impact of material A on keratinocyte function and resilience.

**Figure 8:**
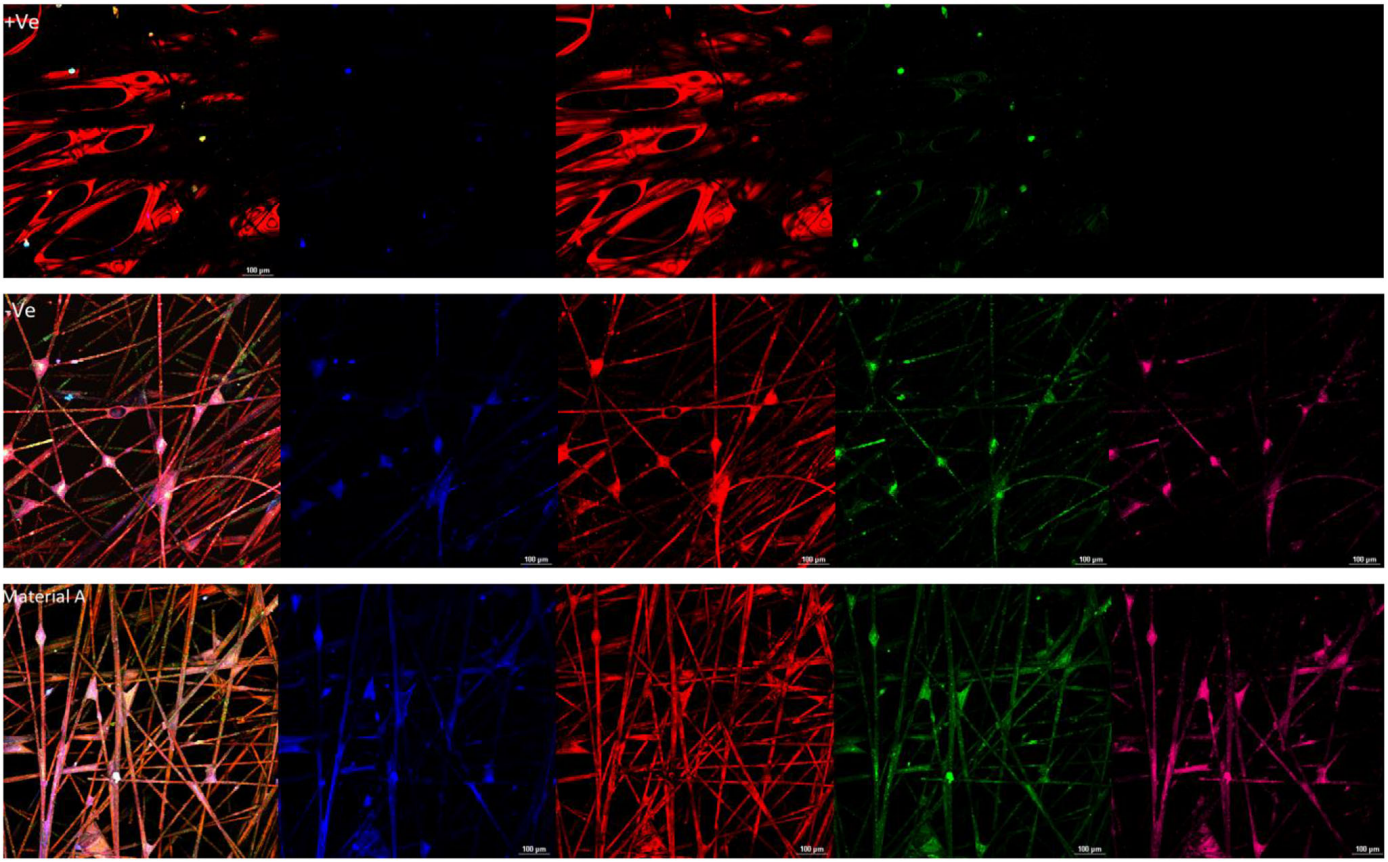
Cytotoxicity analysis after 2 h using a 3D HEKa cell model and confocal microscopy (n = 3) (20x objective). Cytoplasmic membranes (red–orange), nuclei (blue), Cytokeratin 14 (pink), and Cytokeratin 19 (green) were stained. Positive control showed cell damage and low distribution, while negative control showed intact cells and keratin networks. Material A resembled the negative control, indicating no cytotoxic effects after 2 h.

The Distel control shows direct cell damage and low cell density in the HEKa 3D model (**Figure** **8**). The negative control presents a vast distribution of cells, with well‐established keratin networks, presenting no cell damage (Figure 8).

Material A closely resembles the negative control, exhibiting well‐defined cytoplasmic membranes, a structured nuclear network, and vast layers of keratin 19, along with the presence of keratin 14 (Figure 8). The 3D confocal images reveal that a 2 h incubation with Material A did not compromise the integrity of the keratin layers or cellular structures, indicating an absence of direct cytotoxic activity.

## 3 Discussion

Material A, featuring a combination of copper and zinc oxide nanoparticles, exhibits sustained bactericidal, fungicidal, and virucidal activity within just 2 h, falling well below BS EN ISO standards 22196:2011 and 21702:2019. This material is effective against a broad spectrum of clinically relevant pathogens while remaining biocompatible with human skin, demonstrating the exceptional versatility and efficacy of the chosen nanoparticles. Material A demonstrates reproducibility across different printing platforms (data not shown), indicating that the stochastic behavior and distribution of nanoparticles in the 3D resin remain consistent.

Most 3D printing techniques and nanoparticle integration involve depositing or coating a nanoparticle layer on the 3D material, which can lose efficiency over time if damaged.^[^ ^76^ ^]^ The literature lacks information on 3D printed materials with nanoparticles directly incorporated into the material; therefore this work demonstrates a novel and effective approach. Comparing data is challenging due to variations in methodologies, particle sizes, characteristics, morphology, combinations, and integration/coating styles, followed by the lack of usage of neutralization media all of which can affect material activity.^[^ ^77^ ^]^

Sánchez‐Salcedo et al. (2023), silver material directly deposited into a 3D scaffold required up to 6 h of exposure to reduce the *E. coli* count, whereas in this research, 2 h of contact neutralized *E. coli* species.^[^ ^78^ ^]^ Machková et al. (2023) coated 3D materials with silver and copper, found only copper significantly reduced CFU against *P. aeruginosa*,^[^ ^79^ ^]^ and Εkonomou, Soe, and Stratakos (2023) found no significant CFU reduction in *E. coli* biofilms with silver and copper‐coated 3D materials.^[^ ^80^ ^]^ Comparing this, material A shows direct neutralization in 1 h, lowering the bacterial count by five logs and full bactericidal effects in 2 h of contact. Sánchez‐Salcedo et al. (2023) integrated silver nanoparticles into 3D scaffolds,^[^ ^78^ ^]^ showing a 1.80 mm inhibition zone for *E. coli*, whereas material A in this study showed a 5 mm inhibition zone. A study incorporated copper in 3D printing material, achieving similar inhibition zones but requiring high‐cost specialized equipment.^[^ ^81^, ^82^ ^]^ Chen et al. (2023) achieved complete bactericidal effects against *E. coli* with 3% silver nanoparticles,^[^ ^83^ ^]^ whereas in this study, only 1% copper and zinc concentration was necessary for a complete bactericidal effect. Ahmed et al. (2023) achieved rapid antimicrobial activity with 90 wt.% copper, a costly method not directly comparable to this research.^[^ ^84^ ^]^

Material A exhibits a broad‐virucidal effect within 2 h against various clinically relevant DNA and RNA viruses. Luk et al. (2024) reported similar results with Polyhexamethylene biguanide (PHMB) embedded in 3D material, achieving full antiviral activity against the alphacoronavirus 229E virus in 2 h.^[^ ^85^ ^]^ Bilynsky et al. (2022) showed that 3D materials coated with zinc or copper reduced the 229E virus by only 0.426 and 0.129 log, respectively, after 2 h,^[^ ^86^ ^]^ whereas the 3D material with nanoparticles in this research achieved a 4‐log reduction. Results from Machková et al. 2023, where the 3D material was coated with silver nanoparticles, are ambiguous, as in the dry method, the control loss viral titer, but the wet method lowered the viral titer to 2 × 10^4^ from an initial titer of 10^6^ in 1 h of contact.^[^ ^79^ ^]^ In contrast, the results presented for material A demonstrated complete virucidal effects in 2 h across multiple species, suggesting that the combination of copper and zinc nanoparticles provides a superior effect, likely due to nanoparticle size, combination, and surface contact.^[^ ^87^, ^88^ ^]^

Following the antipathogenic results obtained, material A was age‐accelerated for one year to establish the longevity of the antipathogenic properties of the material. The aged materials were tested against two clinically relevant bacteria and one virus. Material A maintained antimicrobial activity and presented better inhibition results compared to the new batch. This difference in inhibition can be based on the oxidation of the nanoparticles during the ageing process,^[^ ^89^, ^90^, ^91^ ^]^ resulting in better activity against the bacterial species tested. No difference in virucidal effect could be observed after material ageing, as the 2 h of contact fully neutralized the influenza virus. This set of results confirms that the material has long‐lasting effects, and the passage of time and environmental factors such as humidity can enhance the bactericidal effects of the 3D impregnated material with nanoparticles. Nanoparticles can change conformation, morphology, or stability based on exposure to the environment, with oxidation and UV exposure playing a major role in the overall behavior of the particles.^[^ ^89^, ^90^, ^91^ ^]^

One advantage of coatings or impregnations is that materials can retain antimicrobial effectiveness for extended periods,^[^ ^51^ ^]^ as observed in material ageing results. It should be noted that various pathogens may develop resistance to surface coatings as an evolutionary response.^[^ ^54^, ^56^, ^92^, ^93^ ^]^ Combining copper and zinc nanoparticles could prevent this development, helping combat the threat of AMR and drug resistance in pathogens. Each metal has a different neutralization mechanism, so resistance to one metal could be countered by the other. While this theory needs further testing, using both nanoparticles together should hinder the development of metal resistance in pathogens.^[^ ^94^ ^]^

Furthermore, the synergetic effect of Cu and Zn may protect copper particles from corrosion and promote copper oxygen reduction through electron displacement, enhancing stability and shelf life. UV light exposure can reverse zinc ageing. UVB irradiation produces an increase in the dissociation process with the oxygen, generating Zn^2+^.^[^ ^95^, ^96^ ^]^ Copper acts as an oxidizing agent, while zinc serves as a reducing agent. Environmental UV light could convert zinc oxide to Zn^2^⁺, zinc, reducing copper oxide back to copper.^[^ ^95^ ^]^ Zinc nanoparticles may protect copper nanoparticles, preserving antipathogenic activity and preventing UV degradation of the 3D material, though further research in this area is needed.^[^ ^97^, ^98^ ^]^

The nanoparticle cytotoxic agar diffusion test showed no significant cytotoxicity or morphological changes in primary keratinocytes after 2 h, with a low reactivity grade of 1 according to BS EN ISO 10993–5:2009. Confocal microscopy with antibodies and cellular dyes revealed no structural or morphological damage in the HEKa cell line exposed to material A, matching the control samples in fluorescence intensity. The 3D scaffolds, mimicking the skin epidermis, showed no cytotoxicity in the 2 h incubation. Cytotoxicity is fully dependent on nanoparticle size, concentration, integration, and dispersion, making direct comparisons with other studies difficult.^[^ ^78^, ^83^ ^]^ For instance, copper nanoparticle toxicity varies widely (30–209.70 µg mL^−1^) in A549/HeLa cells, and zinc toxicity ranges from 25 to 100 µg mL^−1^ in various cell lines.^[^ ^86^, ^89^, ^99^, ^100^ ^]^ Human epidermal cells (HaCaT or A‐375) also show cytotoxic effects dependent on these factors.^[^ ^101^, ^102^ ^]^ Due to the difference in methodology and the nanoparticle characteristics, comparing results may lead to inaccurate interpretations. Both 2D and 3D models indicate that Material A may be safe for up to 2 h of skin contact. However, further in vitro studies are necessary to assess prolonged exposure, along with additional time points. Additionally, in *vivo* investigations, such as long‐term exposure studies in animal models like mice, are required to confirm its biocompatibility over extended durations.

## 4 Conclusion

In conclusion, this research has pioneered an accessible, robust, and replicable method for producing a 3D material impregnated with a nanoparticle mixture (Cu and Zn), which exhibits formidable, long‐lasting antibacterial/antifungal and antiviral properties while maintaining minimal cytotoxicity. Comprehensive screening and rigorous testing in accordance with BS EN ISO standards against a broad spectrum of pathogens, coupled with thorough cytotoxicity assessments, validate its biocompatibility and efficacy. These results suggest that the versatile material A has the potential for safeguarding various environments against pathogenic threats, from crowded public spaces to critical healthcare settings, thereby alleviating time and financial stress associated with housekeeping. This research underscores the material's potentially pivotal role in preventing pathogen infections, reducing transmission rates, and combating antimicrobial resistance. This innovation could represent a crucial breakthrough in our defence strategy against future endemics, epidemics, and pandemics.

## 5 Experimental Section

### 3D printing

The 3D material was created using commercially available Elegoo Standard LCD UV‐Curing resin (SLA) with the Elegoo Mars 2 Pro 2K 3D SLA printer. 3D disks were designed in AutoDesk Inventor (2022 version) as sla file and sliced with CHITUBOX software (version 1.9.3) as a pm3 file. All disks were standardized to a diameter of 0.5 cm and height of 0.2 cm to minimize the random stochastic distribution of the nanoparticles in the 3D material (Figure S1, Supporting Information). Printing parameters were adjusted as detailed in Table S1, Supporting Information)

The resin was mixed and poured into the printer's tank without bubbles. After printing, the material was washed and cured using the ANYCUBIC Wash and Cure 3 Station: washed for 10 min in 99% IMS, air‐dried in a fume cabinet, and UV‐cured for 5 min. This protocol was also tested on the commercially available ANYCUBIC Photon M3 Max SLA 3D printer for replicability, and no notable difference was observed.

### Nanoparticles Selection

Nanoparticles (as detailed in Table S2, Supporting Information) were evaluated, individually or in combination (Figures S2 and S3, Supporting Information), using minimum inhibitory concentration (MIC) and minimum bactericidal/fungicidal concentration (MBC/MFC) assays to determine their effectiveness in solution.^[^ ^65^ ^]^ Based on these initial results, the optimal combinations (copper and zinc oxide 1.12 mg mL^−1^) were selected for 3D material integration (MBC/MFC data are presented for the nanoparticles exhibiting the most optimal results).

### Incorporation of Zinc and Copper Nanoparticles

To prepare the 3D impregnated material with the nanoparticle mixture, 1.5 ml of 25% H_2_SO_4_ was added to 25 ml of SLA resin, mixed, and then 0.5 g of each copper and zinc particles were added to achieve a 1% nanoparticle concentration. This mixture was topped up to a volume of 50 ml, vortexed for 2 min and added directly to the 3D printer tank to create the 3D disks. Negative controls (inert 3D plastic) were fabricated using 50 ml of 3D printable SLA material with 1.5 ml of 25% H_2_SO_4_. The 3D impregnated and control materials were cured and washed as per the printing methods. All assays were conducted in a BSL‐2 biosafety hood to prevent environmental contamination, and the 3D materials were sterilized with 70% IMS prior to use. For simplicity, the 3D impregnated material with the nanoparticle mixture will be referred to as material A.

### Antibacterial/Antifungal Testing

Bacterial and fungal species were supplied from ATCC and NCTC. The pathogens, associated media, and growth conditions are detailed in Table S3 (Supporting Information). All experiments were conducted in biological triplicate with technical duplicates, adjusted at McFarland Standard of 0.5 and following ISO standard BS EN ISO 22 196:2011. SCDLP neutralization media was used to ensure no false positive results were recorded.

### Agar Diffusion Assay

Specimen‐specific plates were pre‐incubated at 37 °C for 30 min. Plates were inoculated using sterile swabs with adjusted overnight cultures, ensuring thorough coverage by swabbing back and forth and then rotating 90°.^[^ ^66^ ^]^ 3D impregnated materials and control materials were placed on the growth media surface, no closer than 24 mm, using sterile forceps. Plates were incubated at pathogen‐specific temperatures, and inhibition areas were measured in cm after 18 h.^[^ ^67^, ^68^ ^]^

### Measurement of Antibacterial Activity on Plastics and Other Non‐Porous Surfaces ISO 22196:2011

BS EN ISO 22 196:2011 measured antibacterial activity on plastics and other non‐porous surfaces. Five hundred microliters of adjusted overnight inoculum were added to material A and control material, covered with a coverslip to prevent evaporation, and incubated at 37 °C for 18 h. The initial inoculum count and recovery rate from the tested materials were established using the Miles and Misra method as CFU mL^−1^.^[^ ^69^ ^]^

Stability and recovery rates were validated according to ISO 22196:2011. The logarithm of the initial specimen count (Lpi) was subtracted from the logarithm of the control material's recovered specimen count after incubation (Lai). A result of 0.2 or less indicated that the reduction was due to material A and not an artefact of the incubation process

### Time Frame of Neutralization via Direct Contact

This assay tests pathogen neutralization time pointed through direct contact with the material.^[^ ^70^ ^]^ Control and material A were inoculated with 500 µl of adjusted overnight culture and incubated at room temperature for both one and 2 h time periods. After incubation, 100 µl of inoculum was added to 900 µl of SCDLP neutralization media, and the Miles and Misra method was then performed. Plates were incubated according to specimen requirements, and results were recorded after 18 h based on bacterial/fungal colony growth to determine the material's antimicrobial or antifungal effects.

### Cell Culture

All cell lines were purchased from ATCC and cultured following the manufacturer's recommendations (Table S4, Supporting Information).

### Virology Assays–Viral Propagation

Viral propagation was conducted based on specimen growth requirements (Table S5, Supporting Information). Harvesting was accomplished when the cytopathic effect (CPE) in cell lines exceeded 60%. Each flask was processed with sterile 2 mm glass beads, followed by centrifugation at 100 g for 10 min. Viral stocks were stored at −80 °C and only used for one freeze‐thaw cycle.

### Viral Titration

Infectious viral titer was determined using plaque assays. After 5 days of incubation, plates were fixed with 4% paraformaldehyde and stained with 1% crystal violet. Viral concentrations were interpreted as plaque‐forming units (PFU).^[^ ^71^, ^72^, ^73^ ^]^

### Antiviral Testing

BS EN ISO 21702:2019 is based on assessing the antiviral activity of plastics and other non‐porous surfaces. Viral experiments were conducted in biological triplicate with four technical replicates. Viral samples, not exceeding 10^6^ PFU mL^−1^, were incubated on material A and control material for 2 h at room temperature in a BSL‐2 cabinet. The recovered titer was then determined using plaque assays.

Viral stability was assessed for two specimens, influenza A and RSV, to ensure no environmental factors influenced viral titer reductions. The viral stability equation was followed as per ISO 21702:2019. The logarithmic difference recorded should not be greater than 0.2, as higher values represented significant titer loss in the incubation process due to environmental factors. No values exceeded 0.2, indicating that the observed differences in viral titer are solely due to the nanoparticles and not an incubation artifact.

### Material Ageing

Material ageing was simulated using accelerated heating, with control and material A subjected to 65 °C for 20 days to mimic one year of ageing.^[^ ^74^ ^]^ After ageing, materials were tested in biological triplicate against MRSA and *E. coli* K12 using the agar diffusion method. The virucidal potency of the aged materials was evaluated against influenza using plaque assays as previously described.

### Cytotoxicity Testing

The BS EN ISO 10993–5:2009 test was used to assess the in vitro cytotoxicity of medical devices based on cell monolayer reactivity. All 3D materials were tested in biological triplicate and technical duplicates using the primary adult human keratinocytes (HEKa) cell line. Indirect contact cytotoxicity was assessed via the agar diffusion method with a modified 2 h incubation. Neat Distel served as the positive control (cytotoxic), and the 3D control material was the negative control. The methodology was followed as per Sandu et al.^[^ ^75^ ^]^ Cell imaging and counting were performed using the Cytation 5 reader with Gen5+ software (version 3.12, Agilent Technologies, Stockport, UK) at ×20 magnification.

### Confocal Microscopy

Confocal microscopy and staining for both 2D and 3D models were performed following the methodology outlined in Sandu et al.^[^ ^75^ ^]^ For 2D models, imaging was conducted using a Nikon A1 LFOV camera mounted on a Nikon Eclipse Ti2‐E microscope. Imaging was performed with a Plan Apo λ 100x oil‐immersion objective, employing laser wide‐field fluorescence with a Galvano scan in a one‐way direction. Image acquisition and analysis were carried out using Nikon NIS‐Elements confocal software.

For 3D models, visualization was conducted using the Nikon Eclipse Ti2‐E microscope with a Nikon A1 LFOV camera, using a Plan Apo λ 20x objective. Imaging parameters included laser wide‐field fluorescence, Galvano scan in a bi‐directional mode, and Z‐axis layer acquisition. The average maximum intensity projection of the Z‐stack was processed and analyzed using Nikon NIS‐Elements software.

### Statistical Analysis

Viral and bacterial data were pre‐processed in accordance with BS EN ISO standards (21702:2019, ISO 22196:2011, and 10993–5:2009). Data analysis, statistical testing, and graphical visualization were performed using Prism software (version 9.5.0). Nikon NIS‐Elements confocal software was utilized for 3D rendering, image analysis, and presentation. All data points are presented as mean ± standard deviation (SD).

Statistical analyses were conducted using Prism software. For cytotoxicity data, an ordinary one‐way ANOVA was performed (n = 3, *p* < 0.05), followed by Dunnett's post hoc test.

## Conflict of Interest

The authors declare no conflict of interest.

## Supporting information



Supporting Information

## Data Availability

The data that support the findings of this study are available from the corresponding author upon reasonable request.
