# Supplementary material for: Bactericidal, Virucidal, and Biocompatible Properties of 3D Printed Materials Enhanced with Copper and Zinc Nanoparticles
Source: Glob Chall. 2025 Jun 16;9(8):e00106. doi: 10.1002/gch2.202500106 (PMC12371212; doi:10.1002/gch2.202500106)
Supplement: Supplementary file 1 — Supporting Information [file GCH2-9-e00106-s001.docx]

**Appendix:**


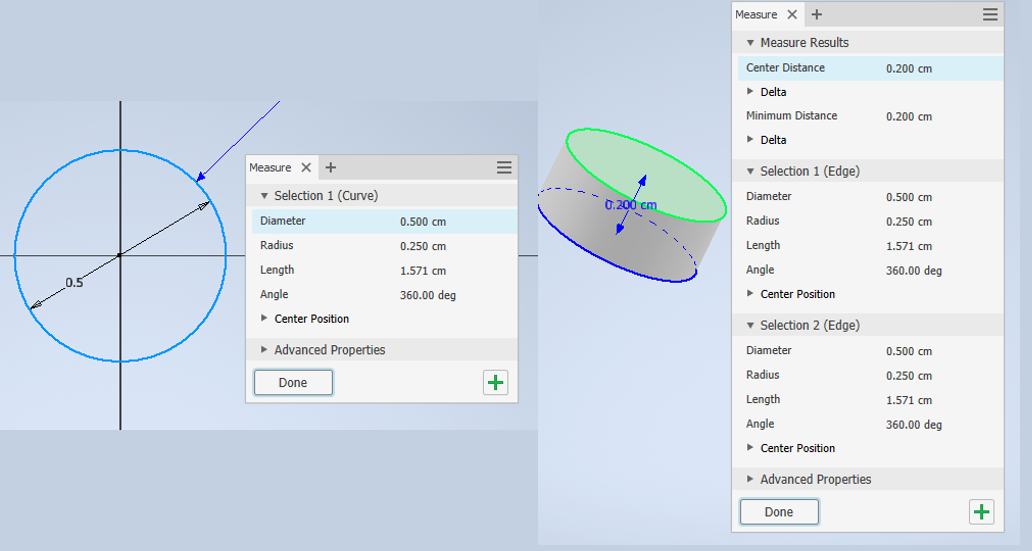


Figure S1. Generation of 3D models. 3D design representation of material disks by using AutoDesk Inventor 2019/2024 software, 2D and 3D structure of the model. Disk diameter of 0.5 cm, radius of 0.25 cm and 0.2 cm height.

| 3D printing | Exposure time | Lift distance | Layer height | Speed |
| --- | --- | --- | --- | --- |
| Bottom time | 80 seconds | 6 mm | 0.05 mm | 180 mm/min |
| Layer time | 12 seconds | 6 mm | 0.05 mm | 180 mm/min |

Table S1. 3D printing parameters. 3D printing conditions, speed and exposure time used for all UV resin 3D printing.

| Nanoparticles | Size | Cat number | Shape or form |
| --- | --- | --- | --- |
| Copper | 25 nm | MERCK 774081 | nanopowder |
| Magnesium oxide | ≤50 nm | MERCK 549649 | nanopowder |
| Platinum nanospheres | 50 nm diameter, 0.05 mg/mL | MERCK 915041 | nanospheres |
| Bismuth (III) oxide | 10 μm | MERCK 223891 | powder |
| Titanium (IV) oxide | <100 nm | MERCK 637262 | nanopowder |
| Zinc oxide | <50 nm | MERCK 677450 | nanopowder |
| Silver nanoparticles | 100 nm | MERCK  576832 | nanopowder |

Table S2. Nanoparticles used for selection via MIC and MBC for material integration. Catalogue number and size of nanoparticles used for stock suspension

| **Name** | **Culture number** | **Conditions for growth on solid media:** |
| --- | --- | --- |
| *Enterobacter cloacae* | NCTC 13406 | Nutrient agar, 24-48 hours, 37°C, aerobic |
| *Enterococcus faecalis* | ATCC 51299 | Nutrient/Blood agar, 37°C, aerobic |
| *Escherichia coli* K12 | NCTC 12923 | Nutrient agar, 37°C, aerobic |
| *Escherichia coli* B | ATCC 23848 | Nutrient agar, 37°C, aerobic |
| *Pseudomonas aeruginosa* | ATCC 27853 | [Trypticase Soy Agar,37°C](https://www.lgcstandards-atcc.org/~/media/F11236DC0E36489ABFA10BF4A411C525.ashx), aerobic |
| *Salmonella enteritidis* | ATCC 13076 | [Nutrient agar or nutrient broth 37°C](https://www.atcc.org/~/media/E6FA2163B72E4DCD880719A2612F2C92.ashx), aerobic |
| *Salmonella typhimurium* | NCTC 12416 | [Nutrient agar or nutrient broth 37°C](https://www.atcc.org/~/media/E6FA2163B72E4DCD880719A2612F2C92.ashx), aerobic |
| *Serratia marcescens* | NCTC 10211 | [Nutrient agar or nutrient broth 37°C](https://www.atcc.org/~/media/E6FA2163B72E4DCD880719A2612F2C92.ashx), aerobic |
| Methicillin Resistant *Staphylococcus aureus* | ATCC 43300 | [Trypticase Soy Agar,37°C](https://www.lgcstandards-atcc.org/~/media/F11236DC0E36489ABFA10BF4A411C525.ashx), aerobic |
| *Candida albicans* | ATCC 76615 | [Potato dextrose agar (PDA) 24°C to 37°C](https://www.atcc.org/~/media/CF1B6EC132F649ED97753F8444E3FE61.ashx), aerobic |
| *Proteus mirabilis* | NCTC BS711 | [Brain Heart Infusion Agar,37°C](https://www.atcc.org/~/media/45E208A3191F4C7EA9B7E1063EB55303.ashx), aerobic |
| *Legionella pneumophila* | ATCC 33152 | Nutrient agar,37°C, aerobic |

Table S3. Bacterial and fungal species. Specimen, culture number and growth conditions

| Specimen | Cell type and origins | Growth Media and CO_2_ | Population doubling |
| --- | --- | --- | --- |
| MRC-5 (ATCC, cat: CCL-17) | Human lung fibroblast- male 14 weeks embryo | EMEM (Corning, cat:10-009-CV) 10%FBS – 5% CO_2_ | Capable of 42 to 46 population doubling |
| Hep2 (HeLa derivative) (ATCC, cat: CCL-23) | Human epithelial- HeLa derived cell line | EMEM/DMEM (Corning, cat: 10-013-CM) 10%FBS– 5% CO_2_ | Immortalised |
| BS-C-1 (ATCC, cat: CCL-26) | African green monkey kidney epithelial | DMEM 10%FBS– 5% CO_2_ | Immortalised |
| A549 (ATCC, cat: CRM-CCL-185) | Human epithelial lung cells- 58 years old male | EMEM/DMEM/RPMI 10%FBS– 5% CO_2_ | Immortalised |
| HeLa (ATCC, cat: CCL-2) | Human epithelial cervix cells- 31 years old female | EMEM/DMEM 10%FBS– 5% CO_2_ | Immortalised |
| MDCK (ATCC, cat: CCL-34) | Canine kidney epithelial cells- adult, female cocker spaniel | DMEM 10%FBS– 5% CO_2_ | Immortalised |
| MA-104 clone 1 (ATCC, cat: CRL-2378.1) | African green monkey kidney epithelial cloned cells | EMEM 10%FBS– 5% CO_2_ | Immortalised |
| A-72 (ATCC, cat: CRL-1542) | Canine fibroblasts- adult female golden retriever, unknown tissue | Leibovitz's L-15 Medium (Sigma-Aldrich, cat: L4386) –atmospheric CO_2_ | Immortalised |
| HEKa | Adult human primary keratinocytes | Dermal cell basal medium (ATCC, cat: PCS-200-030), keratinocyte growth kit (ATCC, cat: PCS-200-040), 5% CO2 | Primary cells, lot dependent |

Table S4. Cell lines used, origins, population doubling and growth media requirements.

| **Name/Species** | **Culture number** | **Growth conditions and host** |
| --- | --- | --- |
| *Betacoronavirus* 1 OC43 | ATCC, cat: VR-1558 | *MRC-5/A549*, EMEM, 0% FBS, 33°C, 3-5 days |
| Human *Alphacoronavirus* 229E | ATCC, cat: VR-740 | *MRC-5/A549*, EMEM, 0% FBS, 35°C, 3-5 days |
| Human parainfluenza virus 3 C 243, HPIV-3 | ATCC, cat: VR-93 | *HeLa*, EMEM/DMEM,2% FBS ,33°C, 2-4 days |
| Human rhinovirus 1A 2060 | ATCC, cat: VR-1559 | *HeLa*, EMEM/DMEM,2% FBS ,33°C, 1-3 days |
| Influenza A virus (H1N1) A/PR/8/34 cell culture adapted PR8 | ATCC, cat: VR-1469 | *MDCK*, DMEM,2% FBS ,37°C, 1-3 days, TPCK 1μg/ml during culturing |
| Rotavirus A Wa | ATCC, cat: VR-2018 | *BS-C-1/MA-104*, EMEM/DMEM,2% FBS ,37°C, 1-2 days, 1-hour activation 37°C with porcine pancreatic trypsin 10 μg/ml (Sigma-Aldrich, cat: T4799), during culturing 2 μg/ml |
| Human respiratory syncytial virus -Long RSV strain | ATCC, cat: VR-26 | *HEp-2* cell, 37°C, DMEM 2% FBS, 3-12 days |
| Human adenovirus 2 | ATCC, cat: VR-846 | *A549* and *MRC-5*, 37°C, EMEM with 2% FBS, 2-3 days |

**Table S5. Viral conditions for each specimen, including catalogue number, cell host, temperature, media, period of infection and supplements necessary for viral propagation.**


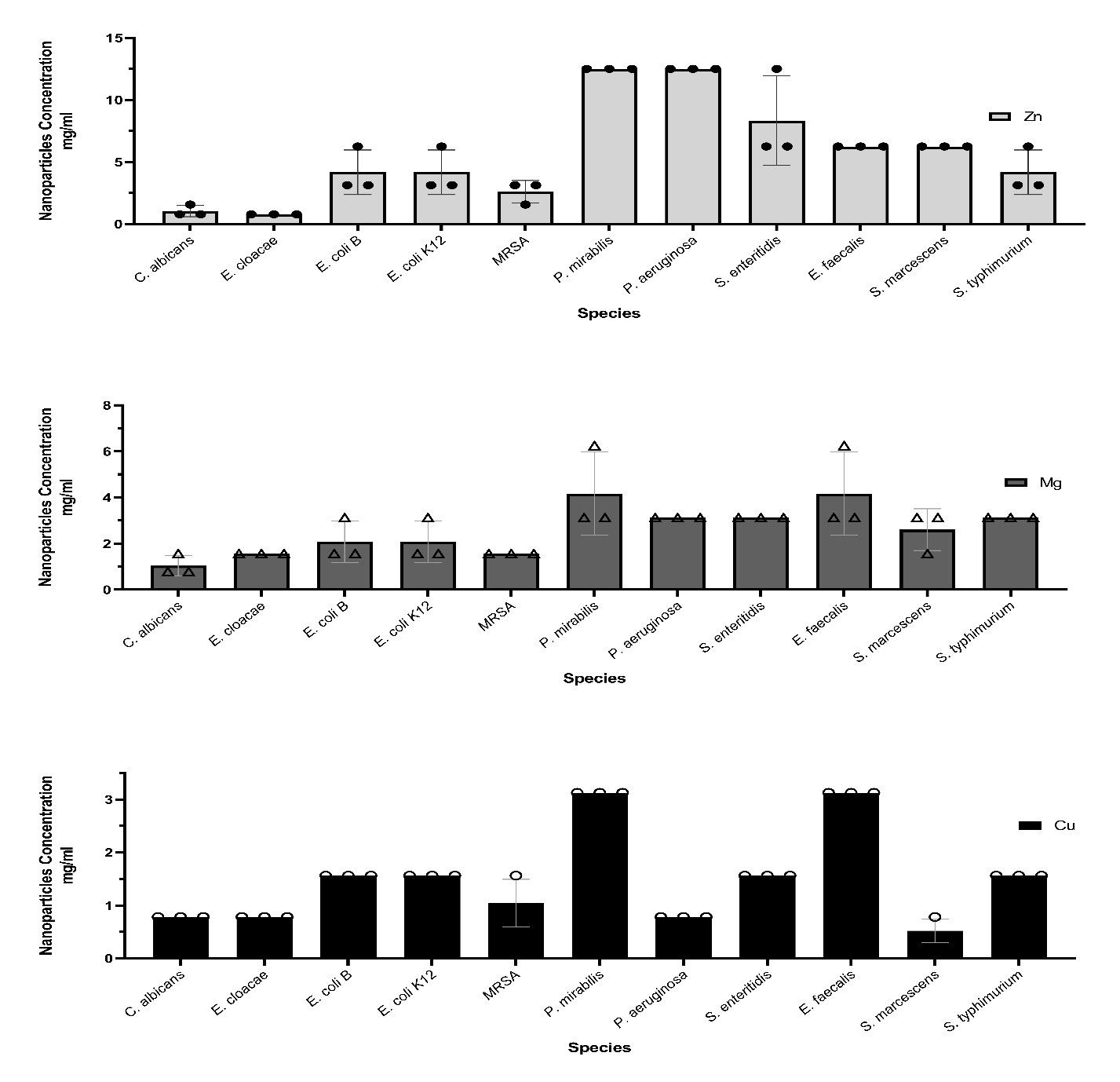


**Figure S2. MBC results for copper, zinc and magnesium nanoparticles represented as mean with SD (n=3) against multiple bacterial and fungal species.**


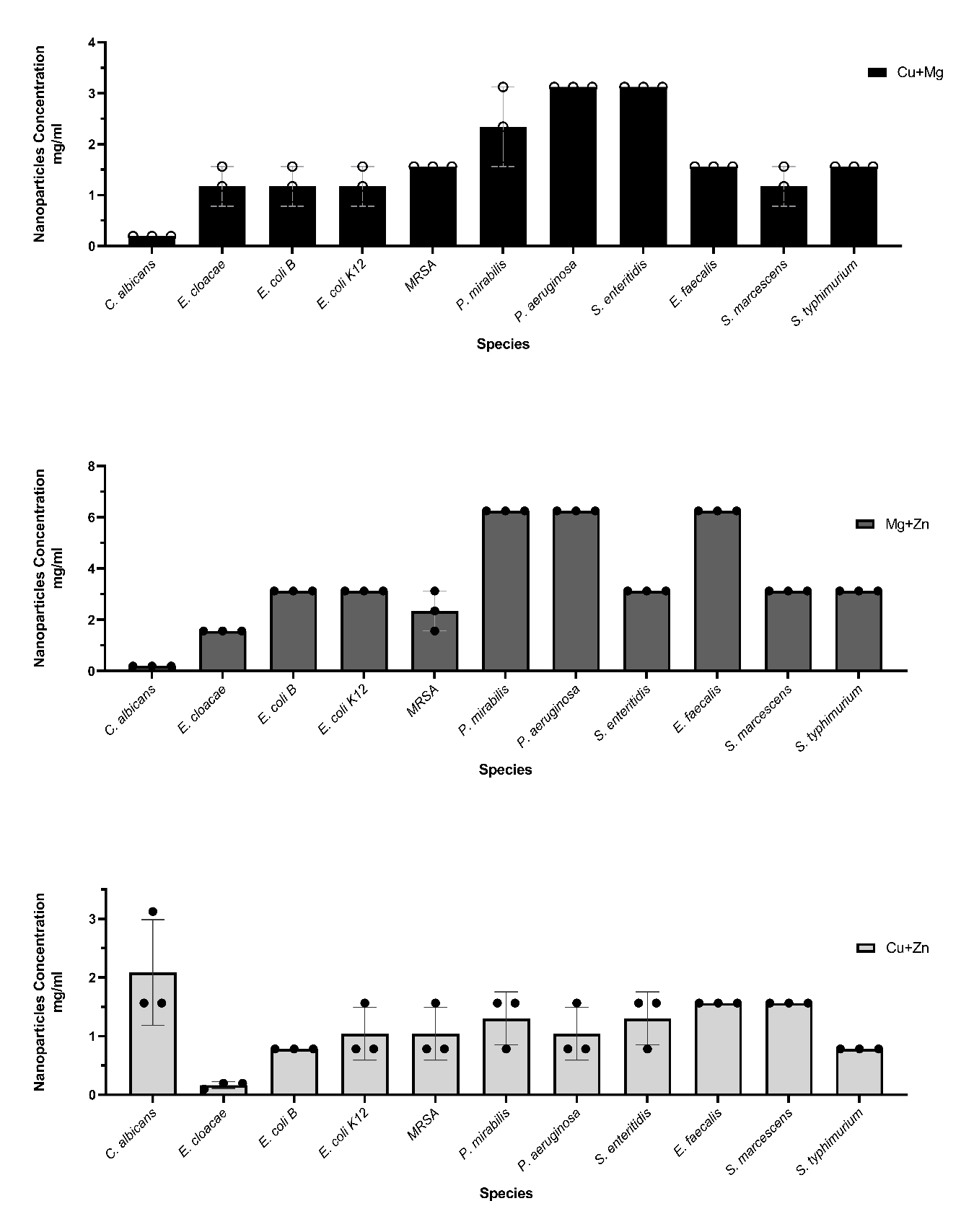


**Figure S3. MBC results for nanoparticles combinations of copper and zinc, copper and magnesium, and zinc and magnesium. Results are represented as mean with SD (n=3).** The most active combination was copper and zinc.

| **Species** | **Common logarithm of bacteria prior to incubation (L_pi_)** | **Common logarithm of bacteria after incubation (L_ai_)** | **Equation results of *(Lpi-Lai) ≤0.2*** |
| --- | --- | --- | --- |
| *C. albicans* | 8.41 | 8.30 | 0.11 |
| *S. enteritidis* | 8.27 | 8.14 | 0.13 |
| *P. aeruginosa* | 8.30 | 8.25 | 0.04 |
| MRSA | 8.55 | 8.50 | 0.05 |
| *E. coli* K12 | 8.50 | 8.50 | 0 |
| *S. marcescens* | 8.47 | 8.44 | 0.02 |
| *P. mirabilis* | 8.20 | 8.14 | 0.05 |
| *E. coli* B | 8.32 | 8.27 | 0.04 |
| *E. cloacae* | 8.20 | 8.17 | 0.02 |
| *S. typhimurium* | 8.46 | 8.43 | 0.03 |
| *E. faecalis* | 8.23 | 8.17 | 0.05 |

Table S6**. The logarithmic difference before and after 18 hours of incubation with the control material.**

**
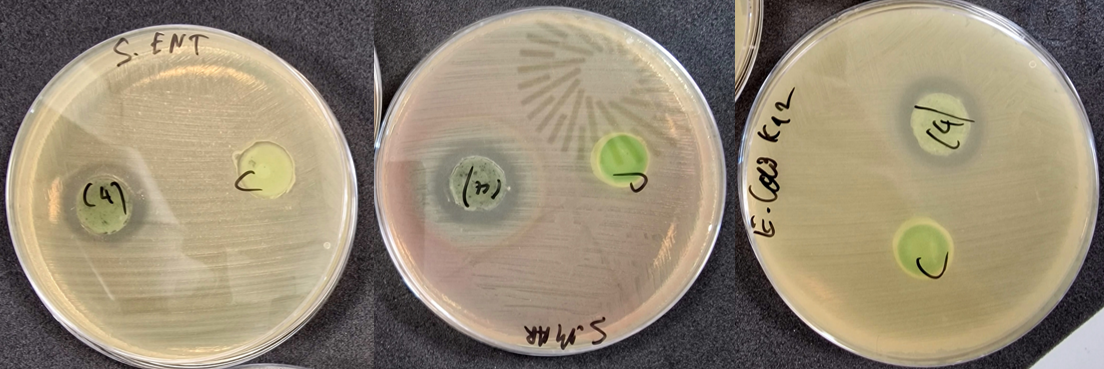
**

**Figure S4. Example of disk diffusion results for material A (n=3), for *S. enteritidis*, *S. marcescens, E. coli* K12.**

**
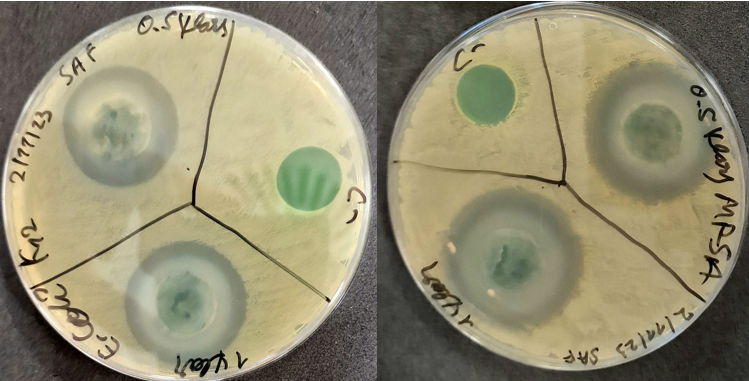
**

**Figure S5. Example of disk diffusion results for material A after ageing (n=3), for MRSA and *E. coli* K12.**
